# New horizons in human sperm selection for assisted reproduction

**DOI:** 10.3389/fendo.2023.1145533

**Published:** 2023-02-22

**Authors:** Brett Nixon, John E. Schjenken, Nathan D. Burke, David A. Skerrett-Byrne, Hanah M. Hart, Geoffry N. De Iuliis, Jacinta H. Martin, Tessa Lord, Elizabeth G. Bromfield

**Affiliations:** ^1^ Priority Research Centre for Reproductive Science, School of Environmental and Life Sciences, The University of Newcastle, Callaghan, NSW, Australia; ^2^ Infertility and Reproduction Research Program, Hunter Medical Research Institute, New Lambton Heights, NSW, Australia

**Keywords:** andrology diagnosis, biomarker, assisted reproductive technologies (ART), male infertility, sperm function assay, sperm

## Abstract

Male infertility is a commonly encountered pathology that is estimated to be a contributory factor in approximately 50% of couples seeking recourse to assisted reproductive technologies. Upon clinical presentation, such males are commonly subjected to conventional diagnostic andrological practices that rely on descriptive criteria to define their fertility based on the number of morphologically normal, motile spermatozoa encountered within their ejaculate. Despite the virtual ubiquitous adoption of such diagnostic practices, they are not without their limitations and accordingly, there is now increasing awareness of the importance of assessing sperm quality in order to more accurately predict a male’s fertility status. This realization raises the important question of which characteristics signify a high-quality, fertilization competent sperm cell. In this review, we reflect on recent advances in our mechanistic understanding of sperm biology and function, which are contributing to a growing armory of innovative approaches to diagnose and treat male infertility. In particular we review progress toward the implementation of precision medicine; the robust clinical adoption of which in the setting of fertility, currently lags well behind that of other fields of medicine. Despite this, research shows that the application of advanced technology platforms such as whole exome sequencing and proteomic analyses hold considerable promise in optimizing outcomes for the management of male infertility by uncovering and expanding our inventory of candidate infertility biomarkers, as well as those associated with recurrent pregnancy loss. Similarly, the development of advanced imaging technologies in tandem with machine learning artificial intelligence are poised to disrupt the fertility care paradigm by advancing our understanding of the molecular and biological causes of infertility to provide novel avenues for future diagnostics and treatments.

## 1 Introduction

Infertility is a relatively common condition afflicting upwards of 15% of couples of reproductive age. Male factors are uniquely responsible for an estimated 30% of couples experiencing difficulty conceiving and beyond this, are a recognized contributor in approximately 50% of all cases of infertility (1). In the four decades since the report of the first successful human *in vitro* fertilization (IVF) (2), considerable gains have been made toward improving the effectiveness of male subfertility treatments. In particular, the advent of gamete micromanipulation techniques, such as intracytoplasmic sperm injection (ICSI), have reframed the field of assisted reproduction by effectively mitigating the need for the fertilizing spermatozoon to exhibit progressive motility, be capable of recognizing an oocyte or completing acrosomal exocytosis (3). Indeed, so profound has the impact of ICSI been in permitting men with defective sperm parameters to reproduce, that it has become the favored choice for fertilization irrespective of the underlying etiology (4). As such, ICSI now features in approximately two thirds of all cycles of assisted reproduction undertaken in countries such as Australia; well beyond that of its initial indication (5). Despite this strategy, worldwide clinical pregnancy and live birth rates resulting from this procedure have remained stubbornly modest at only 26.8% and 20% per initiated cycle, respectively (3). Notwithstanding confounders, recent epidemiological data has also raised concern regarding the prospect of an increased risk of birth defects and the propagation of substandard semen profiles in men conceived using ICSI compared to that of their naturally conceived peers (6, 7). Such findings affirm the need for cautious utilization of ICSI beyond its intended application for men with severely compromised semen parameters and present a strong case for exploring new diagnostic approaches for sperm selection, patient stratification, and therapeutic treatment options for sub/infertile males (8). In this review, we give consideration to current and future directions in male infertility research that are helping address the long-standing question of how to personalize and improve the clinical management of infertile patients.

## 2 Diagnostic andrology

**Figure 1:**
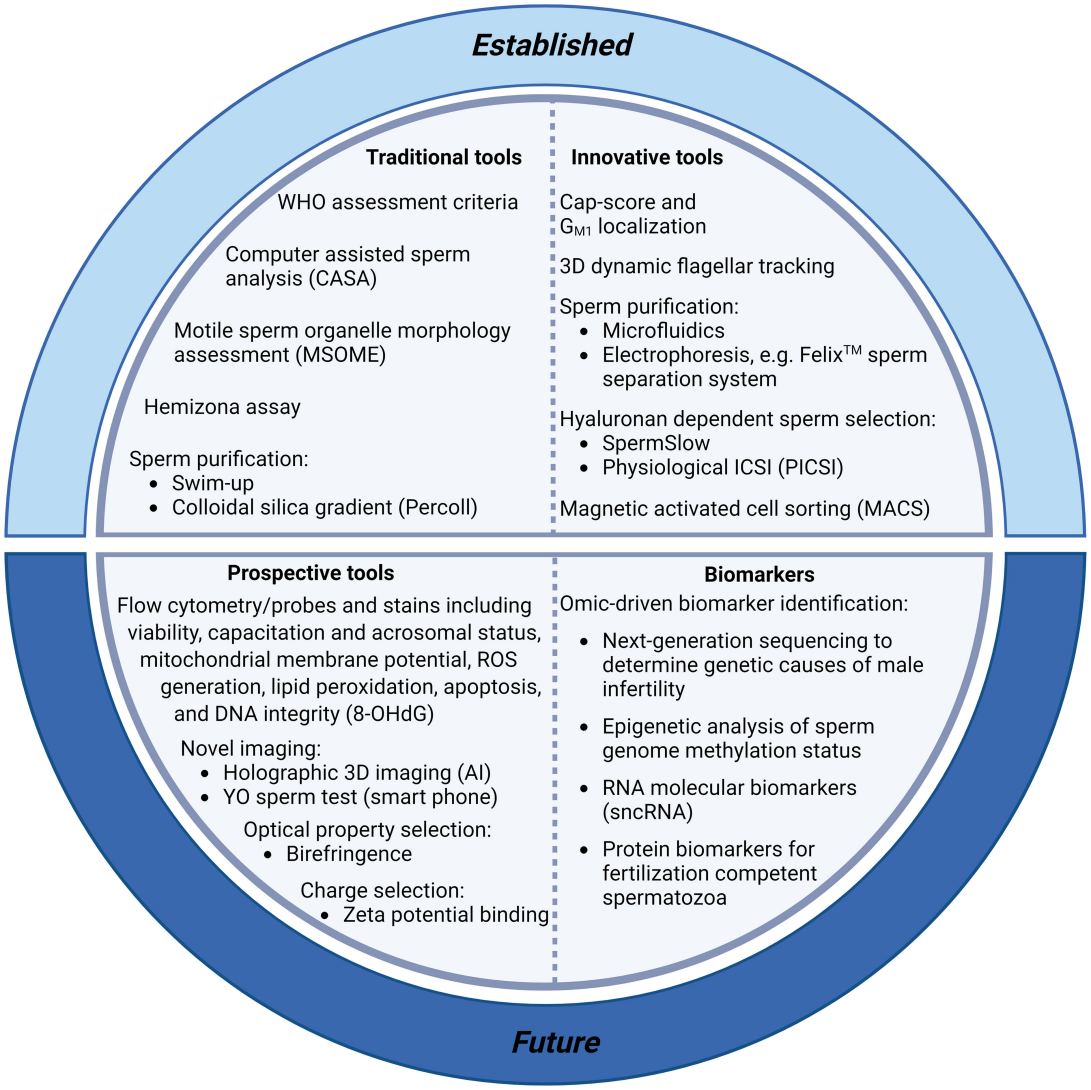
Summary of current and prospective tools for sperm selection and the clinical diagnosis of male infertility. Conventional protocols for the diagnosis of male infertility include assessment of sperm morphology, motility and concentration [including World Health Organisation (WHO) assessment criteria (35), computer assisted sperm analysis (CASA) (17), and motile sperm organelle morphology assessment (MSOME)] (16). These protocols are facilitated by the purification of sperm cells from seminal plasma with swim-up techniques or, more commonly, with density gradient centrifugation based on the use of colloidal silica suspensions. More innovative sperm selection strategies including microfluidics (36) and electrophoresis (Felix™; (37)) are also showing promise for use in clinical settings. Recent additions to the armoury of sperm selection tools include assays with the ability to determine DNA integrity and fertilization capacity [including the Cap-Score test (38), hyaluronan-based sperm immobilization methods (39), and high-throughput flow cytometry assays to detect DNA integrity [e.g., probes for the oxidized base 8-hydroxyl-2-deoxyguanosine (8-OHdG) (40)]. Further, smart phone technology is making semen analyses more accessible with at home YO sperm tests (41), and artificial intelligence (AI) advances have enabled new holographic 3D sperm imaging (28) and sperm quality assessment. Finally, technological advancements in omics platforms (34, 42) are permitting the identification of molecular biomarkers with which to stratify infertile patients, allowing enhanced evaluation of subfertility phenotypes, aiding our understanding of the genetic and epigenetic causes of male infertility, and potentially revolutionising fertility treatment with personalized therapeutic regimens.

Traditional approaches to diagnostic andrology are grounded in the principle that a male’s fertility can be assessed using routine descriptive criteria of the semen profile, with emphasis being placed on sperm count, sperm morphology and sperm motility parameters (**Figure 1**). Unfortunately, this conventional clinical strategy sheds little light on the underlying infertility etiology or the functional integrity of a patient’s spermatozoa and has thus proven to be of limited utility in predicting fertilization success (9). Indeed, despite iterative improvements in the semen assessment guidelines curated by the WHO (10–12), a substantial proportion of infertile men (~15%) still present with ‘normal’ semen profiles according to WHO criteria (13). Such cases of unexplained, or idiopathic, infertility have led us to appreciate that there are many different pathologies that contribute to male sub/infertility, each of which impose different clinical implications for patient management and help account for the relatively weak prognostic value of routine andrological assessment pipelines (14). Similarly, although advances have been made toward the automation of sperm morphology assessment *via* the coupling of ultra-high magnification with machine learning [i.e., ‘motile sperm organelle morphology examination’ (MSOME)], with the goal of standardizing the detection of morphological anomalies, it remains uncertain which elements of sperm structure define the functionality of this highly specialized cell. Thus, despite the potential of improving reproductive outcomes associated with using MSOME in tandem with ICSI (i.e., intracytoplasmic morphologically selected sperm injection; IMSI) (15), there remains insufficient evidence to support a positive effect of IMSI on either clinical pregnancy, live birth or miscarriage rates (16). While such pitfalls could theoretically be addressed by the application of selective stains to discriminate key features of sperm structure and quality (e.g., fluorophores that differentially label spermatozoa according to their viability, acrosomal status, capacitation status, mitochondrial membrane potential, generation of reactive oxygen species (ROS), peroxidation of membrane lipids, apoptosis and the integrity of their DNA), the use of such probes is currently prohibited in clinical practice; and will likely remain so until such time as the development of alternative stains that can achieve the non-destructive labeling of sperm structures in a manner that is either reversible or biologically inert.

As a descriptive stalwart of conventional semen profiling, the assessment of sperm motility offers some promise, especially when this assay is performed with objective computer aided semen analysis (CASA) systems that accurately measure the kinematics of swimming spermatozoa (17). It follows that positive correlations have been reported between the concentration of progressively motile spermatozoa and the outcome of human sperm-cervical mucus interaction tests (18) as well as overall fertility (19). Unfortunately, since the sperm motility profile is not static, such correlations are often weak. Indeed, under the influence of the differing physiological environments that spermatozoa encounter during their passage to the site of fertilization, they variably display forward progressive motility, complete quiescence (permissive of the formation of a storage reservoir in the isthmus of the fallopian tubes), and a characteristic high‐frequency high‐amplitude, asymmetric flagellar beat known as hyperactivated motility (20, 21). Whilst CASA measurements can discriminate these alternate forms of sperm motility (17), their intermittent nature limits their application as robust diagnostic criteria in a clinical setting. This phenomenon also likely contributes to the situation whereby the application of ‘swim up’ techniques to select motile spermatozoa have failed to deliver improved pregnancy rates compared to that of the most widely utilized colloidal silica density gradient preparation methods (22–24). With the intention of providing a more complete appraisal of sperm motility characteristics, new acquisition methods are in development that enable high-resolution reconstruction of three-dimensional sperm trajectories (i.e., *via* real-time tracking of the position of the whole flagellum in three-dimensional space), with some also featuring simultaneous analysis of the morphological characteristics of individual free swimming sperm cells (25–28). One such recently described application exploited a high-speed off-axis holographic system to map the three-dimensional refractive-index profile of the sperm head, in tandem with the dynamic flagellum localization during free swim (29). The four-dimensional reconstructed profile so generated enabled the specimen’s natural movement to be tracked together with detailed volumetric data on internal organelle structures (such as the nucleus housing the paternal genome); all without an attendant need for cell staining (29). Notwithstanding the considerable promise afforded by these new developments, both in the context of biological assays and clinical use, it remains uncertain whether they will be able to resolve the limitations associated with the continuum of motility profiles displayed by the fertilizing spermatozoon.

## 3 Strategies to improve diagnostic andrology

**Figure 2:**
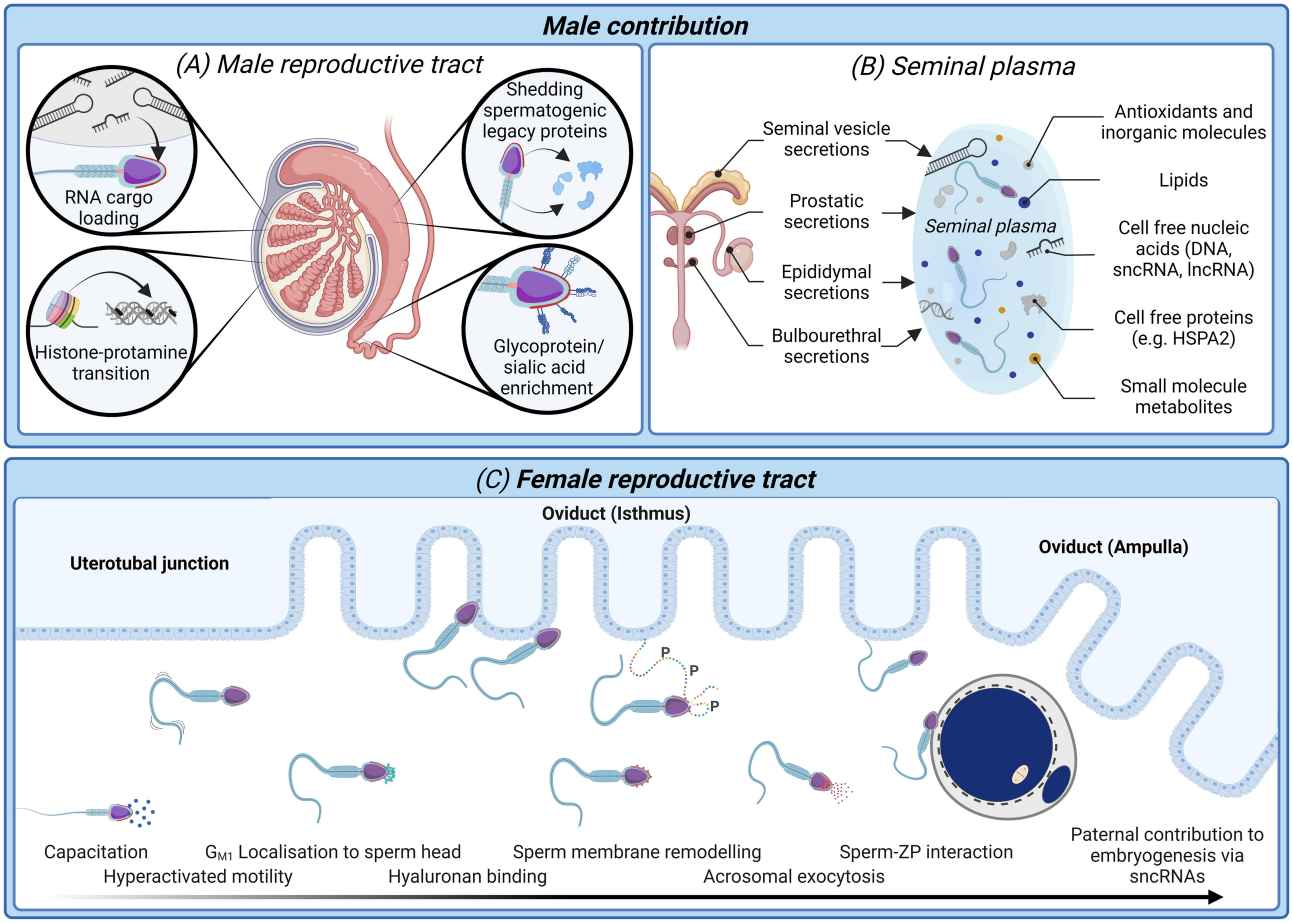
The biological processes and biomarkers of sperm production and functional maturation; an untapped resource for diagnostic tools of male infertility and sperm selection techniques. **(A)** In the male reproductive tract sperm nuclear compaction transitions from histone packaging to one characterized by predominantly protamines (43). Developing spermatozoa are simultaneously loaded with regulatory RNA cargo (42, 44) while also shedding excess cytoplasm and spermatogenic legacy proteins (34). Mature spermatozoa are identifiable upon completion of epididymal transit by an enrichment glycosylated surface proteins (34) and dynamic changes in their small non-coding RNAs (sncRNA) profile (45, 46). **(B)** Seminal plasma conveys not only spermatozoa to the female reproductive tract, but also several non-cellular components including antioxidants, lipids, and cell-free nucleic acids and proteins (47). Many of these non-cellular features offer high potential as biomarkers of a healthy, fertile human ejaculate. **(C)** After spermatozoa are deposited in the female reproductive tract they must successively negotiate the uterus and uterotubal junction before progressing through the oviduct isthmus and ampulla to contact the ovulated oocyte. Thereafter, spermatozoa must undergo a process termed capacitation, which is accompanied by dynamic protein phosphorylation events giving rise to altered patterns of motility (hyperactivated motility) and substantial membrane remodeling to facilitate the presentation/unmasking of the sperm surface receptors that orchestrate cumulus matrix (hyaluronan) and sperm-zona pellucida adhesion and penetration (30, 48). Finally, spermatozoa undergo an acrosome reaction leading to release of their acrosomal contents and remodeling of the sperm head architecture compatible with downstream gamete membrane fusion and fertilization (49). Beyond the delivery of the paternal genome, there is now compelling evidence that sperm convey epigenetic factors including sncRNAs to influence the trajectory of pre-implantation embryogenesis (50, 51).

It is clear that there is a pressing need for new modalities of fertility diagnosis and the selection of spermatozoa for use in ART. In terms of addressing the limitations and variability inherent in current methodologies, we have much to learn from studying both the molecular basis of sperm dysfunction and equally, the features of functionally competent spermatozoa that can successfully ascend the female reproductive tract to reach the site of fertilization and complete the sequence of cellular interactions that precede natural conception (**Figures 1**, **2**). This latter, highly selected subset of spermatozoa not only possess the requisite motility to gain entry into the oviducts but also the competence to engage in capacitation; a complex suite of biochemical and biophysical changes that prime spermatozoa for interaction with the cumulus oophorus and zona pellucida (ZP) surrounding the ovulated oocyte (30). These interactions are orchestrated by specialized membrane domains adorning the anterior sperm head (31, 32), which are formed during spermatogenesis before being extensively modified coincident with sperm transit of both the male and female reproductive tracts (33, 34). It therefore stands to reason that the development of sperm selection protocols that mimic the stringency of the barriers that fertilizing spermatozoa naturally encounter have obvious appeal (**Figure 2**).

### 3.1 Sperm-hyaluronic acid binding

Among an ever-expanding list of emerging sperm selection technologies designed to impose the physiological stringency of natural sperm selection barriers are protocols that exploit the hyaluronic acid (HA) binding properties of spermatozoa. These techniques harness the principle that functionally competent spermatozoa are endowed with HA binding sites that permit their adherence to, and digestion of, the HA-rich cumulus oophorus matrix (52). Two dominant protocols have been marketed under the umbrella of HA sperm selection, i.e., physiological intracytoplasmic sperm injection (PICSI; which features the use of HA immobilized on a solid support) and SpermSlow (which features HA suspended within a viscous medium). Such systems immobilize or retard sperm movement, theoretically enabling the positive selection of mature spermatozoa in preparation for ICSI (52). Notwithstanding the appeal and clinical interest in such interventions, current evidence appears insufficient to support their ability to improve either clinical pregnancy or live birth rates resulting from ART (53–55). Meta analyses have also concluded current evidence is inadequate to exclude adverse effects or differentiate the efficacy of SpermSlow versus PICSI HA binding systems (53). On the contrary, recent re-analysis of data from a parallel, two-group, randomized trial (i.e., HABSelect) has now reported that the use of PICSI is positively associated with a reduction in the rate of miscarriages, particularly in the subset of women aged over 35 (39, 55). Such an effect has been attributed to PICSI selection of spermatozoa with reduced levels of DNA damage.

In terms of offering a biological explanation for the lack of consensus regarding the clinical outcomes of sperm selection achieved using HA-based systems, there is now compelling evidence that the surface architecture of a human spermatozoon undergoes a substantial capacitation-associated remodeling in preparation of oocyte interaction (30, 33, 56–59). This event is characterized by a reduction in the proportion of sperm cells with surface exposed hyaluronidase enzymes [such as sperm adhesion molecule 1, (SPAM1)] (48, 60–62) and a reciprocal increase in those cells presenting ZP receptors on their surface (56). These receptors include arylsulfatase A (ARSA), an enzyme that is predominantly expressed on the surface of capacitated human spermatozoa where it displays affinity for the sulfated sugar ligands adorning the ZP (58, 62, 63). Importantly, recent work has demonstrated that sperm dysfunction may be due to the ubiquitination of ARSA on the sperm surface leading to an altered sperm surface glycosylation profile which, in turn, may negatively impact sperm-oocyte recognition (64). Thus, HA adhesion may favor the selection of the subset of spermatozoa that have yet to complete capacitation. However, whether this delay is reflective of underlying lesions in capacitation-associated signaling remains to be determined. Irrespective, the importance of capacitation as a predictive measure of fertility success is highlighted by recent developments such as the Cap-Score Sperm Function Test (65). As discussed below (please see Section 3.4.3: Sperm lipid biomarkers), this assay is formulated to assess sperm capacitation based on evaluation of the spatial distribution of the dominant sperm ganglioside, G_M1_.

### 3.2 Sperm-zona pellucida binding

After penetration of the HA-rich cumulus oophorus, spermatozoa encounter their final physiological barrier to fertilization in the form of the ZP (**Figure 2**); a glycoprotein matrix that envelops the ovulated oocyte (66). It is well established that an inability to bind and penetrate the ZP ranks among the most common defects recorded in the spermatozoa of infertile male patients (67). Thus, ~10% of men diagnosed with idiopathic infertility present with a failure of sperm-ZP adhesion (68). However, defects in sperm-ZP adhesion are commonly a matter of degree and upon quantification using techniques such as the hemizona assay (HZA), provide the highest discriminatory power for both *in vitro* and *in vivo* fertilization success of any sperm parameter (69). These findings have been taken as putative evidence that the ZP serves an important role in the selection of high quality spermatozoa (33, 66), a notion that resonates with multiple reports that the ZP preferentially binds spermatozoa that display superior motility, morphology, high levels of DNA integrity and lower global DNA methylation levels than that of their unbound counterparts (70–73). In accounting for this phenomenon, it is perhaps notable that each of these functional parameters are acutely sensitive to elevated levels of reactive oxygen species, the excessive production of which is a common etiology in the spermatozoa of infertile males (74). Indeed, work in our own laboratory has shown that molecular chaperone proteins (i.e., heat shock protein A2; HSPA2), which coordinate capacitation-associated membrane remodeling, are highly vulnerable to adduction by reactive lipid aldehydes [including 4-hydroxynonenal (4HNE)] formed as a consequence of oxidative-stress induced membrane lipid peroxidation (75–77). Such adductions disrupt HSPA2-client protein interactions and consequently limit the surface presentation of ZP receptors in capacitated spermatozoa (63).

As an extension of these findings, it has been shown that the use of ZP binding as a tool for selecting spermatozoa for downstream use in ICSI is associated with the production of higher quality embryos as well as improved implantation and clinical pregnancy rates compared to spermatozoa selected by conventional subjective criteria (71, 78, 79). Despite the biological importance of ZP binding, the advent of ICSI to bypass this physiological barrier has effectively diverted attention away from research into the molecular basis by which spermatozoa recognize and engage in productive interactions with the ZP (32). Nevertheless, the balance of clinical evidence as well as that compiled from animal studies indicates that this event likely involves contributions from both protein-protein and lectin-like interactions (80–84). Such observations raise the prospect of using ZP ligands to harvest superior quality, fertilization competent spermatozoa. Although the use of native ZP holds obvious appeal in this regard, ethical and resourcing limitations prohibit the use of this resource. Nevertheless, complex carbohydrate surrogates, such as fucoidan and neoglycoproteins decorated with the sialyl-Lewisx (sLex) sequence, have been identified that possess the ability to competitively inhibit human gamete interactions (81, 85). Similarly, agarose beads coated in recombinant human ZP2 peptides have shown promise in the selection of high-quality human spermatozoa (84). Taken together, these data support the tenant that specific carbohydrate and/or ZP peptide motifs can be harnessed as sperm selection strategies to enhance the success of downstream ART applications. Building on this principle, below we briefly discuss several other advanced sperm selection techniques that are in development based on exploiting the surface characteristics of functionally viable spermatozoa; including those that discriminate sperm on the basis of the degree of negative surface charge, exteriorization of apoptotic markers, and birefringence properties (**Figures 1**, **2**). Notably, however, while several of these technologies have shown promise in a pre-clinical setting, additional high quality randomized evidence is required before advocating for their widespread clinical adoption (53, 54, 86).

### 3.3 Sperm surface characteristics

In common with their somatic cell counterparts, the surface of a human spermatozoon is furnished with a complex glycocalyx comprising a diversity of carbohydrates linked to membrane protein and lipid anchors (87, 88). Moreover, this acellular coat is substantially remodeled during the consecutive stages of sperm maturation, such that those spermatozoa that have successfully completed their epididymal maturation are characterized by an enrichment of glycoproteins with terminal sialic acid residues (89). This gradient of increasing sialylation is now a well-recognized hallmark of sperm maturity and one that has been associated with the protection of spermatozoa from immune surveillance within the female reproductive tract (89). However, beyond masking the allogeneic properties of the sperm cell, the accompanying increase in electronegativity brought about by sialyation has formed the basis of electrophoretic separation techniques designed to rapidly fractionate mature spermatozoa away from other deleterious ejaculate contaminates (i.e., immature germ cells, bacteria, and leucocytes) (90) (**Figure 1**). One such recent development, now marketed under the trademark of the Felix Sperm Separation System, has shown promise for the isolation of suspensions of viable, morphologically normal spermatozoa with high levels of total and progressive motility, and low levels of DNA damage (37, 90–93). Clinical compatibility has also been demonstrated with biopsied material, snap-frozen sperm suspensions and cryostored semen, and the first report of a human pregnancy and live birth using electrophoretically isolated spermatozoa (91). Alternate electrokinetic properties of mature spermatozoa including the ~ -16 to -20 mV charge differential across their plasma membrane (i.e. their zeta potential) have also been exploited as a means by which to capture these cells following their adherence to positively charged support matrices (94). Akin to electrophoretic separation methods, methods exploiting zeta potential have been reported to select mature sperm cells with enhanced levels of normal morphology, kinematic parameters and DNA integrity, and accordingly, have been associated with improved fertilization and pregnancy success following ART (95).

Among the leading non-genetic etiologies causally linked to male infertility is oxidative stress, a pathology that arises as a consequence of elevated levels of reactive oxygen species (ROS) within the male germline (74). Sperm cells are highly vulnerable to oxidative stress owing to an abundance of oxidizable substrates (including high levels of polyunsaturated fatty acids) combined with the fact that they possess limited capacity to protect themselves against oxidative attack or to enact repair once they sustain damage (74). Instead, spermatozoa revert to an apoptotic cascade upon the induction of oxidative stress, a situation that eliminates their fertilization potential and presumably promotes their phagocytosis without the propagation of an attendant inflammatory response within the female reproductive tract (96). One of the late hallmarks of the apoptotic sperm membrane is the externalization of phosphatidylserine resides, the detection of which forms the basis of negative selection protocols, including glass wool separation and magnetic activated cell sorting (MACS)-annexin V technologies, designed to remove senescent spermatozoa from within an ejaculate prior to ART (53, 54, 97–100). One potential flaw in this otherwise laudable approach is that the superficial exposure of phosphatidylserine has recently been reported in the head region of viable and motile mouse spermatozoa (101). Indeed, the surface presentation of phosphatidylserine is reported to progressively increase during sperm transit through the epididymis and again upon the capacitation of these cells. Moreover, the masking of sperm surface phosphatidylserine residues potently inhibited fertilization, thus identifying this ligand as a potential key player in sperm-oocyte fusion (101). Such findings raise the prospect that the loss of phospholipid asymmetry within the sperm plasma membrane is not simply a signature of apoptotic cells but rather one associated with the level of sperm functional maturity. As an important caveat however, it remains to be determined whether this model also holds true in the case of human spermatozoa. It is thus premature to conclude whether this is an extenuating factor that has limited the clinical utility of MACS-annexin V selection protocols (53, 54).

Apart from measurable differences in the macromolecular composition of the sperm surface that herald changes in their functional status, maturing sperm cells are also characterized by changes in their birefringence optical properties. Birefringence is a phenomenon exhibited by certain materials in which an incoming (incident) ray of light is split by polarization into two rays each assuming slightly different paths and travelling at different velocities. The extent of this so called ‘bi’refraction (i.e., birefringence) is determined by the non-uniform spatial (i.e., anisotropic) properties of the material; which in the case of human spermatozoa, originate from a combination of longitudinally orientated protein filaments residing within the subacrosomal, nuclear, and axonemal domains, each of which refract light differently (102–104). Accordingly, the use of polarized light microscopy has drawn attention as a diagnostic tool with which to evaluate the structural integrity of spermatozoa with applications extending to the selection of viable cells with normal morphology and low levels of DNA damage in preparation for ICSI (105, 106). However, the clinical utility of this technology has yet to be investigated in randomized trials (53).

### 3.4 Sperm biomarkers

As discussed above, there are numerous inherent limitations associated with existing strategies for sperm selection and defining male fertility. These limitations have, in turn, fueled renewed interest in the identification of non-invasive biomarkers that accurately predict fertilization outcome and provide molecular insight into underlying infertility pathophysiology (107). Such approaches have been aided by rapid technological advances in analytical ‘omic’ platforms leading to a recent proliferation in studies seeking to define the macromolecular signatures (e.g. lipidomic, metabolomic, proteomic, and epigenomic) of semen and spermatozoa from fertile males versus those produced by subfertile/infertile men (57, 108–112).

#### 3.4.1 Sperm protein biomarkers

Due to their inability to engage in *de novo* protein synthesis, the functional maturation of human spermatozoa is reliant on changes associated with two phases of post-testicular maturation, namely: (i) the incorporation of new proteins encountered during their descent through the male reproductive tract (epididymis), and (ii) the processing and/or post-translational modification of their intrinsic proteome that occurs during post-ejaculatory capacitation (33, 34, 57) (**Figure 2**). These characteristics highlight the importance of proteomic tools for studying the molecular changes that drive the acquisition of sperm functionality (57, 110, 111). It follows that considerable research effort has been directed to cataloging the sperm proteome in addition to the seminal fluid and innumerable extracellular vesicles in which they are bathed (108, 109, 111, 113–116). In the context of spermatozoa, this growing proteomic resource has been assembled into a comprehensive reference library of 6,198 proteins; an inventory that accounts for ~80% of the estimated 7,500 proteins that are represented in human spermatozoa (117). Notably, recent work from our own laboratory has demonstrated that alongside proteins that are either gained and/or modified during post-testicular sperm maturation, the shedding of proteins also constitutes a major part of the proteomic changes that contribute to producing a fertilization competent sperm cell (34). Such proteins, which are underrepresented in the mature spermatozoon, have been shown to map to several infertility phenotypes and could thus represent negative selection markers to potentially identify poor quality spermatozoa. Amid the remaining challenges to realizing the transformative impact of this information are the investigation of protein interaction networks, characterization of those proteins whose function is influenced by post-translational modifications (e.g. phosphorylation, proteolytic cleavage, acetylation, glycosylation), and defining anomalies in protein abundance that signify specific defects in sperm function (57, 109).

Advances are being made toward these goals through the application of advanced mass spectrometry technology platforms to deliver new insight into the proteomic features of spermatozoa in different functional states (i.e., fertile *vs*. infertile, immature *vs*. mature, and non-capacitated *vs*. capacitated) (34, 61, 118–122). Such strategies are helping to shed light on specific proteomic elements that are of functional significance and facilitating improved interpretation of the spectrum of post-translational modifications necessary for generating a fertilization competent spermatozoon (57). Illustrative of this potential, mass spectrometry based proteomic analyses have been used to identify specific defects in human sperm cells associated with their failure of ZP recognition (61). Among the subset of proteins significantly under-represented in the spermatozoa of infertile patients, this study identified the molecular chaperone, HSPA2 (61). Such unbiased findings align with independent evidence that the overall abundance of HSPA2 in human spermatozoa provides a robust discriminative index of their fertilizing potential (52). By way of a biological explanation, HSPA2 appears to be a critical component of the machinery that orchestrates sperm plasma membrane remodeling events during spermiogenesis and capacitation (56, 59, 62, 123–125). Thus, an under-representation of HSPA2 likely has functional consequences in terms of the functional priming of ZP binding domains on the mature sperm surface. Moreover, the under-representation of HSPA2 within the sperm proteome has been causally linked to oxidative stress (63), thus reinforcing the concept that the fidelity of sperm-ZP interactions can be used to sensitively monitor the legacy of this pathophysiological challenge.

Aside from HSPA2, a subset of other sperm proteins have been identified as targets for damage brought about by the formation of oxidative 4HNE adducts (126–128), including A-kinase anchor protein 4 (AKAP4); a sperm-specific protein that localizes to the fibrous sheath of the flagellum, where it fulfils indispensable roles in spermatogenesis and subsequently in the support of sperm motility, capacitation-associated signaling and chemotaxis (129, 130). Data from our own laboratory have shown that both the AKAP4 protein and its precursor (proAKAP4), are targeted for 4HNE adduction in primary cultures of round spermatids and in mature mouse and human spermatozoa (127). We further demonstrated that exogenous 4HNE challenge of round spermatids and spermatozoa leads to a significant reduction in the detectable levels of both proAKAP4 and AKAP4 and a concomitant compromise of capacitation-associated phosphotyrosine expression in human spermatozoa; the latter putatively being caused dysregulation of the signaling network assembled around the AKAP4 scaffold. Such data affirm the utility of proAKAP4/AKAP4 as markers of sperm function and identify the measurement of proAKAP4/AKAP4 abundance as a promising approach to evaluate semen quality in male infertility disorders (131–133).

#### 3.4.2 Sperm nucleic acid biomarkers

In addition to sperm proteins, alternative macromolecular features such as the sperm RNA signature and the integrity of the paternal genome have also attracted attention as prospective markers of fertilization success (134, 135). With regard to the latter, it has long been of interest to understand the principles, and biological consequences, associated with the packaging of approximately two meters of DNA into the sperm nucleus to create a structure far more compact than that of its somatic cell counterparts. It is now understood that this remarkable feat is accomplished by substantial remodeling of the chromatin during spermiogenesis linked to replacement of the majority of DNA packaging histones with protamines; effectively reducing the paternal DNA to a quasi-crystalline state approaching the physical limits of compaction (136) (**Figure 2**). In addition to streamlining the morphological profile of the spermatozoon, this strategy has attendant consequences in terms of reducing the exposure of the paternal genome to damaging agents yet effectively silencing transcription such that human spermatozoa possess limited capacity to enact DNA repair if they sustain damage to the paternal genome (137, 138). When attacks to the paternal genome do occur, they are commonly oxidatively induced as evidenced by the formation of the 8-hydroxy-2’-deoxyguanosine (8OHdG) DNA base adduct (139–141). Notably, such lesions are not randomly dispersed across the genome in mature spermatozoa but rather are targeted, with chromosome 15 being particularly susceptible to oxidative stress (142); a phenomenon potentially linked to its position within the nuclear matrix (143). Moreover, areas of vulnerability to oxidative attack are commonly associated with inter-linker regions (142); short (<1000 bp) non-protein bound segments interspersed between protamine-bound DNA toroids (43, 144, 145) and retained histone-bound DNA units that affix DNA to the nuclear matrix (146–148). Notably, these regions of vulnerability are of clinical relevance owing to the fact that they have been shown to harbor oxidative damage in the spermatozoa of infertile male patients (142). It follows that such defects (in addition to those associated with genetic mutations, exposure to environmental agents and disruption of chromatin proteins) have been causally linked to natural reproductive failures including reductions in fertilization rates, pregnancy rates, embryo quality, and increased rates of spontaneous abortion (149–151). However, it remains uncertain to what extent the evasion of natural conception barriers by directly injecting spermatozoa with damaged DNA into an oocyte, accounts for the increased risk of birth defects associated with assisted conception technologies such as ICSI (7, 152–154). Moreover, the retention of histones in approximately 4-15% of the sperm nuclear genome, contributes to a scenario in which epigenetic marks can endure reprogramming events (138, 155) thereby enabling transcriptional memory with potential implications extending across generations (156).

Accordingly, a battery of diagnostic assays have been brought to market that evaluate sperm DNA fragmentation such as the sperm chromatin structure assay (SCSA) (157), the single cell gel electrophoresis (Comet) assay (158), the terminal deoxynucleotidyl transferase mediated deoxyuridine triphosphate nick end labeling (TUNEL) assay (159), the chromomycin A3 test (160), *in situ* nick translation (161), the sperm chromatin dispersion (SCD) test (162, 163), and protamine ratio analysis (164). Regrettably, none of these report on the epigenetic state of the genome, and similarly, current evidence linking sperm DNA damage with outcomes of ART interventions is controversial. Thus, although some literature reports a strong negative association between sperm DNA fragmentation and pregnancy outcomes to justify the incorporation of sperm DNA testing into routine clinical tests (165, 166), other systematic reviews and meta-analyses suggest that our current armory of DNA damage assays have limited ability to predict pregnancy outcomes in the context of ART (167, 168). Thus, despite the potential benefits, there remains insufficient impetus to foster the routine application of sperm DNA damage testing in the management of couples seeking recourse to ART. We do, however, advocate for the continuation of high-quality research into the predictive value of sperm DNA fragmentation assays for both the success of pregnancy and for the choice of ART treatment.

Beyond the implications of sperm DNA damage and histone modifications, several classes of regulatory RNA species have also been implicated in epigenetic inheritance, including multiple sub-types of sperm-borne small non-coding RNAs (sncRNAs). Indeed, despite their transcriptionally and translationally inert state, it is well documented that mature spermatozoa convey a heterogenous cargo of RNA transcripts, including, mRNA, long non-coding RNA (lncRNA), and sncRNA (169). Moreover, a subset of the latter have been linked with regulation of the trajectory of pre-implantation embryo development and consequently, influencing the lifetime health of offspring (170). Thus, rather than being insignificant vestiges of spermatogenesis, sperm-borne sncRNAs are gaining attention for their potential prognostic value in evaluating sperm quality linked to male infertility (169). Growing interest has also focused on sperm-borne sncRNAs as diagnostic markers of a male’s exposure to environmental stressors and the fidelity of sperm production (134, 169). In this regard, mounting evidence has drawn links between an array of environmental stressors (e.g. dietary challenges, stress hormone administration, imposition of psychological stress, and exposure to environmental pollutants such as cigarette smoke) and pronounced alterations in the sperm sncRNA profile of exposed males (51, 171, 172). Of concern is that each of these changes can potentially contribute to sperm dysfunction and may account, at least in part, for the observation that the sperm sncRNA signature of idiopathic infertile males is evidently different from that of fertile individuals (169, 173–176). Notwithstanding the interest these studies have generated, the distinctive features of sperm biology pose several hurdles to utilizing RNA cargo for diagnostic purposes. Key among these are the low yield and the highly degraded nature of sperm RNA, yet it is hoped that these limitations will, in time, be circumvented by continued optimization of RNA isolation protocols (177). Such advances are necessary to realize the translational potential of data collected from pre-clinical animal studies and ensure the reproducibility of clinical assessments of male fertility using RNA biomarkers. Ultimately, the cataloging of human sperm RNA, similar to that achieved for the sperm proteome, may herald new opportunities to assess the effects of environmental, physical and chemical factors on semen quality with a level of unparalleled sensitivity.

#### 3.4.3 Sperm lipid biomarkers

As integral components of the sperm surface, the composition, orientation and distribution of membrane lipids have also attracted attention as diagnostic biomarkers of male fertility. Illustrative of this potential are the MACs sperm selection technologies described previously, which exploit the externalization of phosphatidylserine in moribund cells (discussed above). However, recent developments have also seen a renewed focus on alternative lipids such as monosialotetrahexosylganglioside (G_M1_), which features as the target of assays marketed under the trademark of the Cap-Score Sperm Function Test (178, 179). In principle, Cap-Scores provide an indication of the percentage of capacitated spermatozoa within a given sample as determined by the localization of G_M1_; an integral component of sperm membrane rafts and one that becomes progressively concentrated within the vicinity of the anterior domain of the sperm head as capacitation proceeds (178, 180, 181). It follows that the re-localization of G_M1_ differs in the spermatozoa of cohorts of fertile and potentially infertile men, giving credence to the utility of Cap-Score. Indeed, Cap-Score has shown promise in predicting the success, or failure, of intrauterine insemination (IUI) in both retrospective (178) and prospective contexts (38, 65). Notably, Cap-Scores appear highly reproducible among ejaculates from a single individual and reliably identify those cells competent of completing an acrosome reaction induced by either an ionophore (179) or progesterone challenge (182); yet they have limited relationship with conventional semen analysis criteria (178). These data identify the utility of the Cap-Score as a predictive measure of male fertility and, if borne out across larger cohort studies, a strategy with important clinical applications in patient stratification. For a comprehensive summary of lipids that may be further developed as biomarkers of male infertility see (112, 183).

### 3.5 Seminal plasma biomarkers

Beyond the sperm component of the ejaculate, the seminal plasma in which they are bathed is also recognized as comprising a rich variety of biomolecules; many of which could potentially serve as non-invasive biomarkers of a male’s fertility status [e.g. proteins (125, 184, 185), small-molecule metabolites (186), lipids (187), cell-free nucleic acid (DNA, sncRNA and lncRNA) (188–191), as well as antioxidant agents and inorganic chemicals (ions) (192)]. Indeed, seminal plasma is an elaborate nutrient-rich fluid generated by the accessory glands of the male reproductive tract, which constitutes as much as 95% of human semen (193). Contrary to the long-held belief that seminal plasma acts solely as a transport medium for spermatozoa, a compelling body of data now supports key physiological roles in the promotion of early pregnancy success (47, 194). Such functions are attributed to direct communication with the female reproductive tract at insemination, effectively modulating cellular, molecular and immunological adaptions of the uterine environment to accommodate the semi-allogeneic conceptus and optimize pregnancy outcomes (47, 194). While it is apparent that human conception can proceed in the absence of seminal plasma, emerging clinical evidence (195–197) in tandem with data arising from pre-clinical animal models (198–201) suggests that an absence of seminal plasma exposure at conception results in suboptimal embryonic development and placentation; changes that can, in turn, manifest in pathological consequences for offspring. Such findings have prompted mounting interest in the active signaling factors present within seminal fluid (47) as well as the susceptibility of these factors to paternal stressors (202–204). Moreover, beyond those molecules with putative roles in modulating the female response, it is emerging that the components of seminal plasma may bear witness to the legacy of defective sperm production. Illustrative of this potential is HSPA2, a molecular chaperone protein previously discussed in relation to its role in the capacitation associated remodeling of the sperm surface, and one that is intimately associated with spermatogenesis. Recent work has also documented the presence of sperm-free HSPA2 in seminal plasma wherein its relative abundance is positively correlated with spermatogenic status (125). It follows that sub-classes of infertile males, such as cryptozoospermic patients, have low to non-detectable seminal plasma HSPA2; likely reflective of an almost total meiosis arrest. Additional work toward characterizing the molecular composition of seminal plasma and its relationship with a male’s fertility status is certainly warranted to help realize the potential of this easily accessible suspension as a source of biomarkers capable of predicting the potential success or failure of ART procedures.

## 4 Implications for therapeutic interventions

With ongoing limitations associated with the accurate diagnosis of male infertility, it is not surprising that a majority of the therapeutic interventions that have been clinically successful have been targeted to the treatment of pathologies with an obvious phenotype. Examples of these successful strategies lie in the treatment of varicocele through surgical means (205), the use of aromatase inhibitors in patients with abnormal testosterone-to-estradiol ratios (206), and extensive developments in ART procedures, such as ICSI (3), that have assisted patients with poor semen parameters, or those with an absence of sperm in the ejaculate through a combination of testicular sperm extraction (TESE) and ICSI.

In the case of varicocele, although the prevalence of the condition remains high at up to 20% of the male population, clinical varicocele now ranks as the most common correctable cause of male infertility (205). Varicocele repair is aimed at the dilation of the pampiniform plexus, a venous network located in the spermatic cord, to relieve the causative retrograde blood flow through the internal spermatic vein. In current practice, surgical approaches to repair varicocele, including ligation and resection of the dilated vessels by open surgery or microsurgery, are preferred. Further, the ability to tailor interventions to the grade and condition of each patient has led to a large array of treatment options (205). These innovations have had a significant impact on the recovery of fertility in patients that have undergone varicocelectomy. However, complications due to the inflammation and testicular heating paradigms associated with this condition have led to the suggestion that varicocele may be a progressive pathological condition, with implications extending to both structural and functional damage within the testis (207). Particularly concerning is that even in varicocele patients with normal semen parameters, or those with improved fertility post-surgery, the condition is commonly accompanied by an increased prevalence of sperm DNA fragmentation (208). Thus, issues associated with reduced paternal DNA integrity may persist in embryos generated through either natural conception or through ART interventions after varicocelectomy.

Although the nature of the sperm DNA damage experienced in varicocele patients is not entirely understood, one hypothesis is that excessive production of ROS in varicocele testes may contribute oxidative DNA lesions in spermatozoa and/or damaged chromatin in developing germ cells (205). One option for the management of these patients, pre- and post-surgery, is the administration of oral antioxidants to reduce the presence of ROS. While there is obvious theoretical appeal to this strategy and sound evidence from recent animal models that report the efficacy of targeted antioxidant strategies to reduce DNA damage (209), the use of oral antioxidants in a clinical setting has been met with mixed success. Recent reviews have highlighted just five oral antioxidant supplements that have resulted in increases in clinical pregnancy rates and live birth rates (76, 210), namely: astaxanthin, L-carnitine in combination with L-acetyl carnitine, zinc sulphate, vitamin E, and Menevit. Despite the initial promise of these therapeutic candidates, there have been few reports regarding the longevity of this success or follow up studies on the consistency of improved pregnancy outcomes following administration of these antioxidants. Moreover, throughout the analysis of 29 clinical studies of antioxidants targeted to men experiencing fertility problems, extensive variation in outcomes was observed with some studies reporting profound improvement in several semen parameters and some reporting no effect using the same oral antioxidant (210).

Certainly, some of this variability can be attributed to disparities in the intrinsic design of clinical trials, with potential confounders including variations in dose regimens, methodology and the duration of treatment. However, recent reports have also highlighted a lack of selectivity in the patient populations that are recruited for each trial (210, 211). Regrettably, very few antioxidant trials have been performed with cohorts of patients specifically selected based on the presentation of oxidative defects in their spermatozoa or high levels of ROS in their ejaculate. Moreover, the measures of success for these trials, while encompassing important outcomes such as improved semen parameters and time-to-conception, do not commonly include assessments to ensure a complete resolution of ROS levels or DNA damage in the patient’s spermatozoa (210). This has led to a concerning inability to account for why individual antioxidant trials have not been successful and also compromises our future ability to improve on formulations that may be beneficial in stratified patient cohorts.

The reasons for omitting crucial patient selection procedures are undoubtedly complex, though difficulties in the diagnosis of ROS-driven fertility issues and the accurate quantification of oxidative DNA damage remain major clinical roadblocks. Here, despite extensive validation of a number of reliable biomarkers for lipid peroxidation products, ROS, and oxidative DNA damage (211), there are still major challenges associated with the cost-efficiency and accuracy of these tools. Consideration should be given to the development of these common laboratory tools to form clinically applicable markers that can be employed for the high throughput analysis of ROS and oxidative DNA damage in human spermatozoa. Working toward this goal, validation has now been performed across several protocols to assess human DNA oxidation levels using 8-hydroxy-2’-deoxyguanosine (8-OHdG) antibodies in tandem with flow cytometry to discriminate patients with poor semen quality (40). Indeed, this study has helped to establish a consensus for a clinically applicable protocol that allows for the stratification of patients based on 8-OHdG fluorescence. Moreover, great care has been taken to provide evidence of the repeatability and accuracy of the assay, and a rationale for its use as part of routine diagnostics in ART clinics (40). It remains to be seen whether the clinical application of this technique will aid in the selection of patients for oral antioxidant trials. However, this approach is a step towards the development of better management strategies for patients with oxidative-stress derived infertility.

The examples provided here serve to highlight the necessity of a continuum between accurate diagnostics to stratify male reproductive pathologies and the successful development of appropriate treatment and management strategies. While there are many exceptions to this rule, such as the management of patients with non-obstructive azoospermia for which there are no current treatment options despite the clarity of the condition, gaining an advanced mechanistic understanding of male reproductive pathologies is essential to improve diagnostic and therapeutic strategies. In this context, the field of reproductive science stands to benefit from diagnostic innovations and personalized treatment strategies that are currently disrupting the standard of care for other clinical pathologies. Indeed, the current key gap in activity regarding precision medicine approaches to optimize ART commits couples to a standard trial-and-error treatment paradigm that is both inefficient and costly. The case for improved treatment selection tools is only further justified by ongoing research and big data efforts, which continue to uncover potential new fertility biomarkers, but we remain some way from actualizing these into novel therapeutic strategies. While most activity is currently focused on female infertility, notable recent efforts discussed above to address male infertility include the introduction of the Cap-Score to assess sperm capacitation, curation of big data arising from the application of omic-based approaches, broader adoption of sperm DNA fragmentation analyses earlier in diagnostic workups, novel imaging tools (e.g. holographic 3D imaging) that leverage artificial intelligence (AI) to improve the accuracy and speed of gamete selection for ART, and at-home smartphone-based semen testing (e.g., the FDA-approved YO Sperm Test).

## 5 Conclusions

Despite males accounting for a substantial proportion of human infertility, we currently lack the tools to accurately diagnose and treat this distressing condition. Traditional diagnostic approaches often overlook the subtleties associated with day-to-day variations in sperm production and have increasingly been found to be inadequate predictors of fertilization outcome and live birth rates. These shortcomings underscore an urgent need to develop improved diagnostic tools to more accurately inform patient stratification, improve sperm selection and identify valid therapeutic treatment options for males afflicted with subfertility and infertility. In terms of realizing these ambitious goals, we have much to gain from continued research into the molecular mechanisms that govern normal sperm function and an improved understanding of how sperm cell biology becomes dysregulated in infertile males. In particular, dissection of the highly specialized sequence of changes that accompany sperm production and their functional maturation during their transit of the male and female reproductive tracts promises to yield novel insights into how these cells behave during *in vitro* interventions (**Figures 1**, **2**). Moreover, with the growing realization that poorer-quality sperm may impact offspring health, we have an obligation to define those contributions of the fertilizing spermatozoon that limit the possibility of an adverse outcome after ART interventions. The development of specific sperm biomarkers for this purpose remains a significant goal as does defining the biological signatures indicative of the stress(ors) that the male may have experienced; information that may eventually provide additional clinical decision support to guide ART treatment strategies and maximize success rates.

## Author contributions

BN, JS, NB, DS-B, HH, GD, JM, TL and EB contributed to conception and design of this article. BN and EB wrote the first draft of the manuscript. JS, NB, DS-B, HH, GDI, JM, and TL each wrote sections of the manuscript. All authors contributed to manuscript revision, read, and approved the submitted version.
